# Supplementary material for: Reversible modulation of superconductivity in thin-film NbSe2 via plasmon coupling
Source: Nat Commun. 2024 Jul 18;15:6037. doi: 10.1038/s41467-024-50452-4 (PMC11255238; doi:10.1038/s41467-024-50452-4)
Supplement: Supplementary file 1 — Supplementary Information [file 41467_2024_50452_MOESM1_ESM.pdf]

Supplementary Information for

Reversible Modulation of Superconductivity in Few-Layer $NbSe_2$ via Plasmon Coupling

Guanghui Cheng[1,2,*], Meng-Hsien Lin[3], Hung-Ying Chen[3], Dongli Wang[4], Zheyan Wang[1], Wei Qin[1,*], Zhenyu Zhang[4,5], Changgan Zeng[1,4,5,*]

[1]CAS Key Laboratory of Strongly Coupled Quantum Matter Physics, and Department of Physics, University of Science and Technology of China, Hefei 230026, China.

[2]Advanced Institute for Materials Research (WPI-AIMR), Tohoku University, Sendai 980-8577, Japan.

[3]MetaSERS TECHNOLOGY Corp., Hsinchu 30264, Taiwan.

[4]International Center for Quantum Design of Functional Materials (ICQD), Hefei National Research Center for Physical Sciences at the Microscale, University of Science and Technology of China, Hefei 230026, China.

[5]Hefei National Laboratory, Hefei 230088, China.

*Corresponding authors. Emails: cheng.guanghui.c2@tohoku.ac.jp; qinwei5@ustc.edu.cn; cgzeng@ustc.edu.cn.

Table of Contents

Supplementary Figure 1. Schematic of the AuNPs capped with 1-octadecanethiolate layer.

Supplementary Figure 2. Schematics of the transport configuration under light illumination.

Supplementary Figure 3. Modulation of superconductivity with and without AuNPs.

Supplementary Figure 4. Superconductivity modulation of device A1 (AuNPs/hBN/$NbSe_2$) with different illumination wavelengths.

Supplementary Figure 5. Replotted typical *R*-*T* curves of device A1 (AuNPs/hBN/$NbSe_2$).

Supplementary Figure 6. Replotted T_c modulation coefficient (unit: Kcm^2/mW) as a function of illumination wavelength.

Supplementary Figure 7. FDTD simulation results.

Supplementary Figure 8. Dependence of T_c of $NbSe_2$ on the nonequilibrium temperature T_1^*.

Supplementary Figure 9. Theoretical analysis of experimental results on the plasmon-modulated superconductivity temperature.

Supplementary Figure 10. Voltage–current (*V*-*I*) characteristics of device A1 (AuNPs/hBN/$NbSe_2$) on a linear scale.

Supplementary Figure 11. Temperature dependent resistance related to BKT physics.

Supplementary Figure 12. Differential conductance spectra of $NbSe_2$ at different photon fluxes and schematic of the electron transport at the normal metal/superconductor interface.

Supplementary Figure 13. Estimation of the temperature rise by COMSOL Multiphysics software.

Supplementary Figure 14. Temperature dependences of the superconducting gap Δ and critical current I_c obtained in AuNPs/hBN/$NbSe_2$ device A3.

Supplementary Figure 15. Sample resistance of device A1 (AuNPs/hBN/$NbSe_2$) as functions of temperature and perpendicular magnetic field *B* (a) without and (b) with plasmon excitation ($N = 1.56\times10^{13}$ $s^{-1}mm^{-2}$).

Supplementary Figure 16. Plasmonic superconducting switch operates in low-bias-current mode.

Supplementary Note 1. Detailed transfer processes of AuNPs.

Supplementary Note 2. Theoretical model of the superconductivity modulation by plasmon.

Supplementary Note 3. Differential conductance and BTK model.

Supplementary References

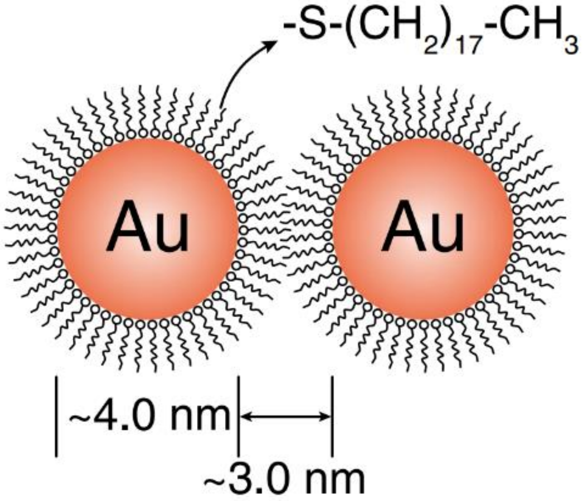

Supplementary Figure 1. Schematic of the AuNPs capped with 1-octadecanethiolate layer. Through the self-assembly process, a monolayer of close-packed AuNPs is prepared on the surface of the $NbSe_2$ device. The diameter of the nanoparticle is ~4 nm and the gap between nearest nanoparticles is ~3 nm.

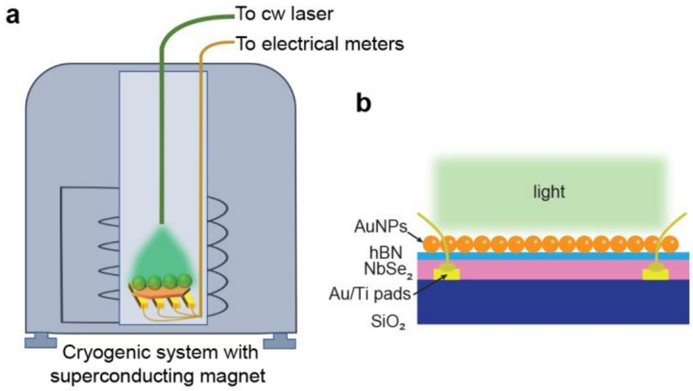

Supplementary Figure 2. Schematics of the transport configuration under light illumination. (**a**) Transport measurements of a device under light illumination in a cryogenic system. The images and all elements are created by the authors. (**b**) Schematic of the AuNPs/hBN/$NbSe_2$ device under light illumination.

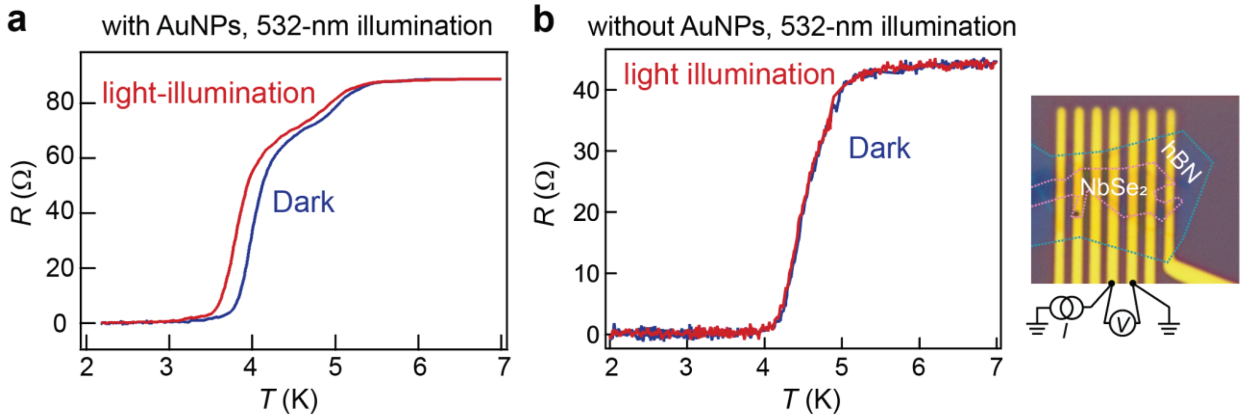

Supplementary Figure 3. Modulation of superconductivity with and without AuNPs. (**a**) Superconductivity modulation of a trilayer $NbSe_2$ device A1 (AuNPs/hBN/$NbSe_2$). The 532-nm photon flux is 1.56×10^{13} $s^{-1}mm^{-2}$. (**b**) Superconductivity modulation of a trilayer $NbSe_2$ device B2 (BN/$NbSe_2$) without depositing AuNPs. The 532-nm photon flux is 1.31×10^{13} $s^{-1}mm^{-2}$. No noticeable modulation of superconductivity is observed. Right panel: the optical image of device B2 with a two-terminal measurement configuration. A contact resistance R_c = 90.5 Ω has been deducted.

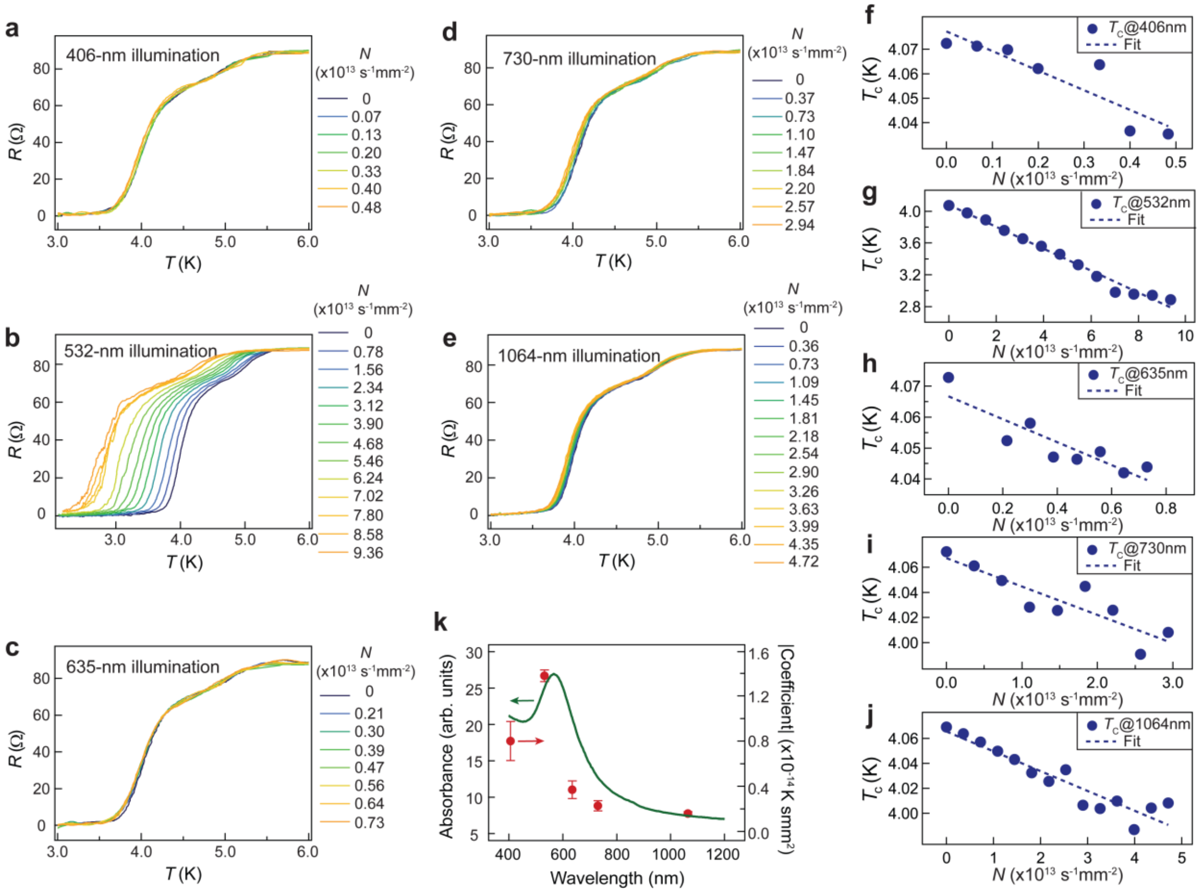

Supplementary Figure 4. Superconductivity modulation of device A1 (AuNPs/hBN/NbSe₂) with different illumination wavelengths. (**a-e**) Sample resistance R as a function of temperature T under light illumination of different wavelengths. The measurements are taken with two-terminal configuration, same to Fig. 1 in the main text. A contact resistance R_c = 54.5 Ω has been deducted. (**f-j**) The critical temperature T_c versus photon flux N for light illumination of different wavelengths. The blue points are experimental results obtained from **a-e**. The dashed lines are linear fittings, whose slopes are defined as modulation coefficients of T_c. (**k**) modulation coefficient of T_c as a function of illumination wavelength. The five data points are obtained from the linear fittings in **f-j**. The error bars are obtained by the linear fittings. The green curve denotes the light absorbance of AuNPs.

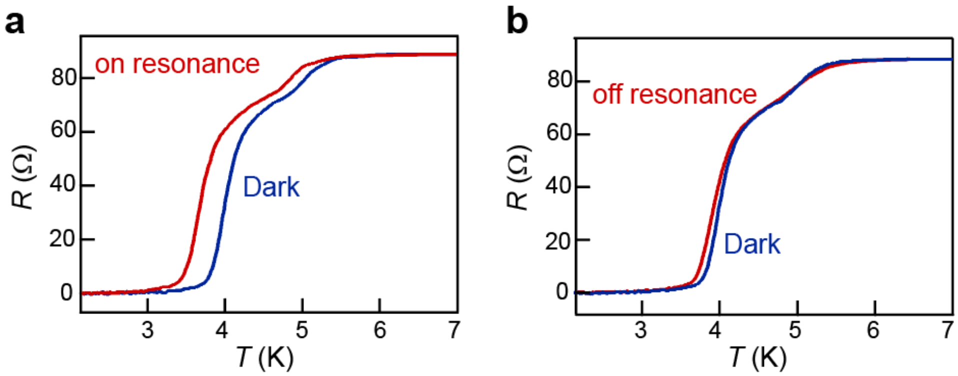

Supplementary Figure 5. Replotted typical *R*-*T* curves of device A1 (AuNPs/hBN/$NbSe_2$). The illumination powers for both (**a**) on resonance and (**b**) off resonance cases are 0.88 mWcm^{-2}.

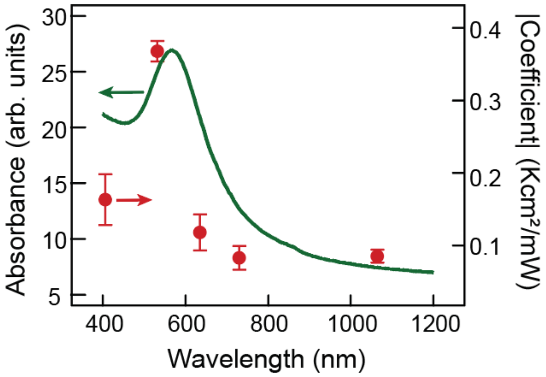

Supplementary Figure 6. Replotted T_c modulation coefficient (unit: Kcm^2/mW) of device A1 (AuNPs/hBN/$NbSe_2$) as a function of illumination wavelength. The error bars are obtained by the same method as in Supplementary Figure 4k.

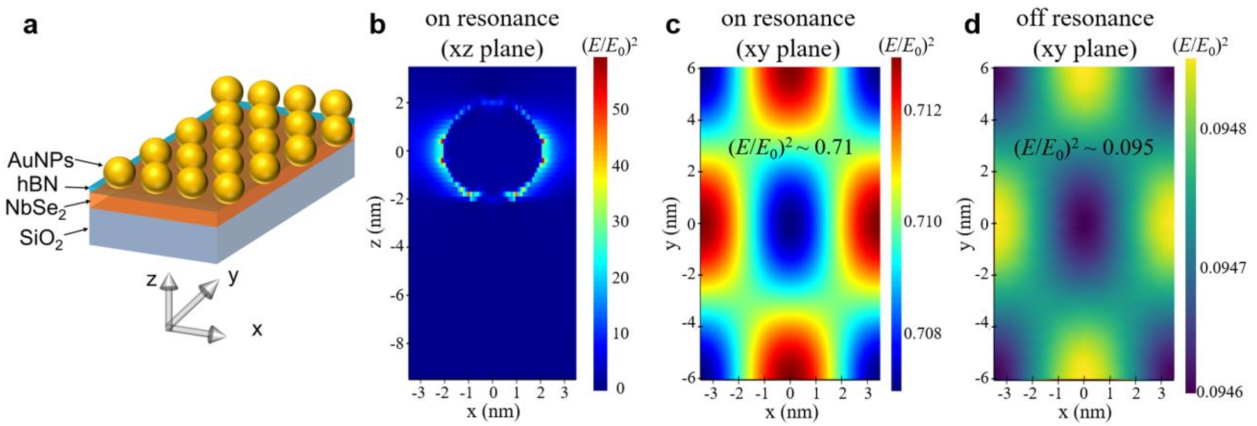

Supplementary Figure 7. FDTD simulation results. (**a**) The device configuration for FDTD simulation. Note that we use a gold film to replace $NbSe_2$ layers to simplify the issue involving the optical properties of superconductors. This is based on the similar electromagnetic response of superconductors and normal metals when the electromagnetic energy surpasses the superconducting gap[1]. (**b**) The electric field strength distribution $(E/E_0)^2$ along the xz plane for the section of typical gold nanoparticle. E_0 denotes the far field of the propagation light. Although the strength of the evanescent field decays fast when getting far away from the surface of the particle, we note that the evanescent field can still have finite response up to tens of nanometers away[2]. (**c,d**) The electric field strength distribution $(E/E_0)^2$ along the xy plane close to the $NbSe_2$ layers (detection is set at z=-6.5 nm) for on resonance and off resonance cases. The blue areas in **c** and **d** correspond to the positions directly below the gold nanoparticles. The red areas correspond to the positions directly below the gaps of the nanoparticles. The dimensions of the simulation are 7×12.1 nm^2, including one periodic structure of AuNPs array with the underlying infinite-size trilayer $NbSe_2$. The light illumination power E_0^2 is unified as 1. To facilitate comparisons with free-space light intensity, the simulation strength of the evanescent field is normalized as $(E/E_0)^2$. The substrate SiO_2 is included in the simulation.

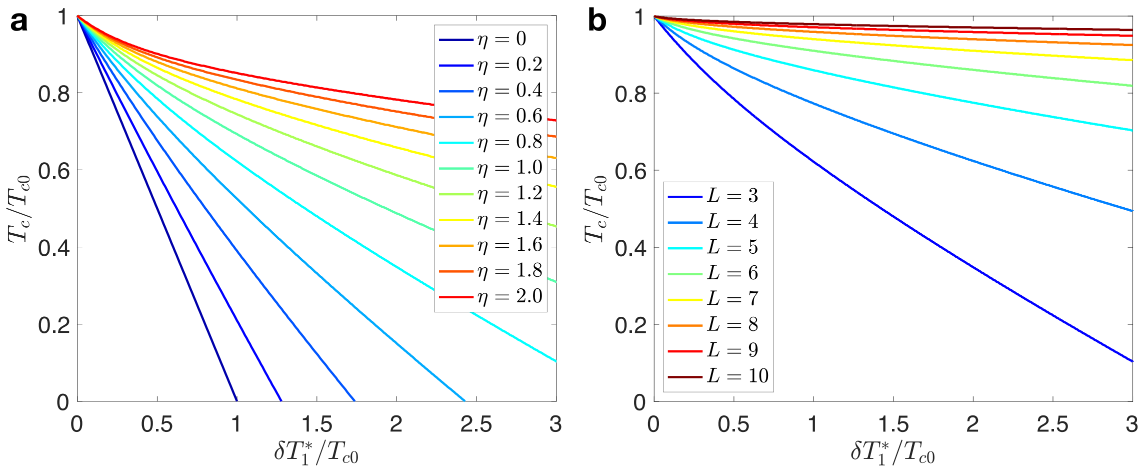

Supplementary Figure 8. Dependence of T_c of NbSe₂ on the nonequilibrium temperature T_1^*. (**a**) Critical temperatures T_c's for 3 layers of NbSe₂, where η is the dimensionless decay coefficient of the plasmon-induced evanescent field, T_{c0} denotes the superconducting critical temperature in a thermal equilibrium state (in the absence of light), and the longitudinal axis $\delta T_1^* = T_1^* - T$ measures the departure of the top layer of NbSe₂ from its thermal equilibrium state. (**b**) T_c as a function of δT_1^* for different thicknesses of the NbSe₂, where L denotes the total number of layers of NbSe₂. We note that T_{c0} in **b** corresponds to the layer-dependent thermal equilibrium critical temperature, which is given by Eqs. (5) and (6). The results shown in **b** are obtained by choosing $\eta = 0.8$.

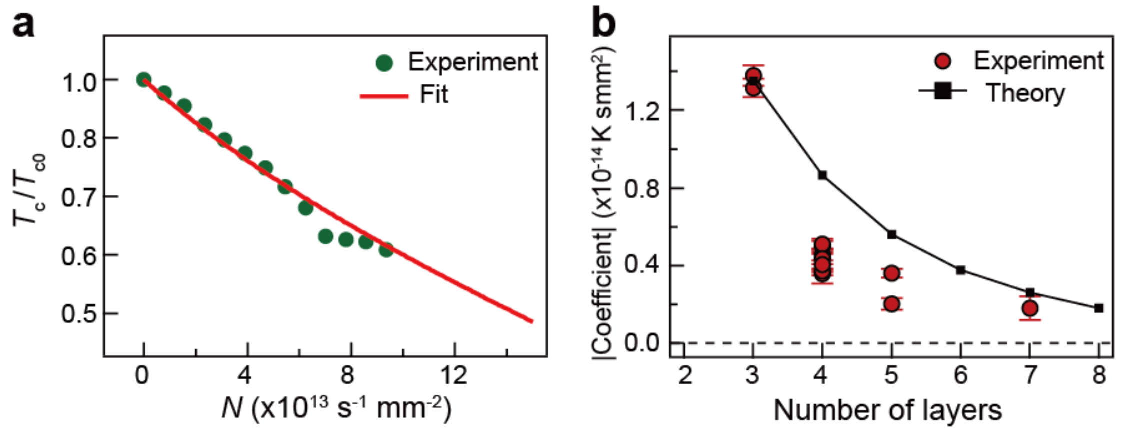

Supplementary Figure 9. Theoretical analysis of experimental results on the plasmon-modulated superconductivity temperature. (**a**) Theoretical fitting to experimental data of T_c (device A1) for $L = 3$ with the parameters $\eta = 0.8$ and $\lambda = \frac{\delta T_1^*}{N} = 3.05 \times 10^{-14}$ Ksmm^2. (**b**) Theoretical prediction and experimental data of the linear suppression coefficient for different thicknesses of NbSe₂. The theoretical prediction is obtained assuming identical parameters for different thicknesses $\eta = 0.8$ and $\lambda = \frac{\delta T_1^*}{N} = 3.05 \times 10^{-14}$ Ksmm^2. The error bars are obtained by the same method as in Supplementary Figure 4k.

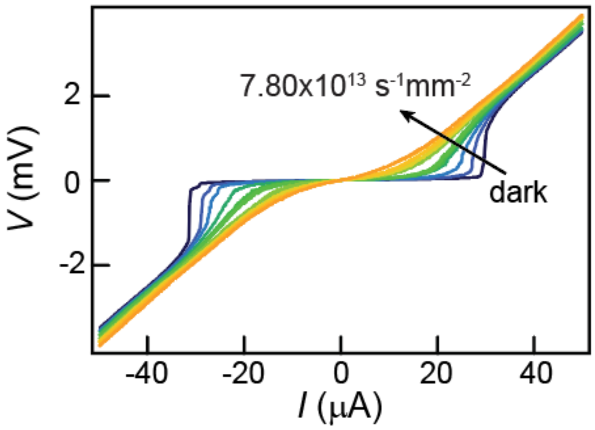

Supplementary Figure 10. Voltage–current (*V-I*) characteristics of device A1 (AuNPs/hBN/$NbSe_2$) on a linear scale. The data are obtained at various photon fluxes, at a temperature of 2.2 K. The measurements are taken with two-terminal configuration, same to Fig. 1 in the main text. A contact resistance R_c = 54.5 Ω has been deducted.

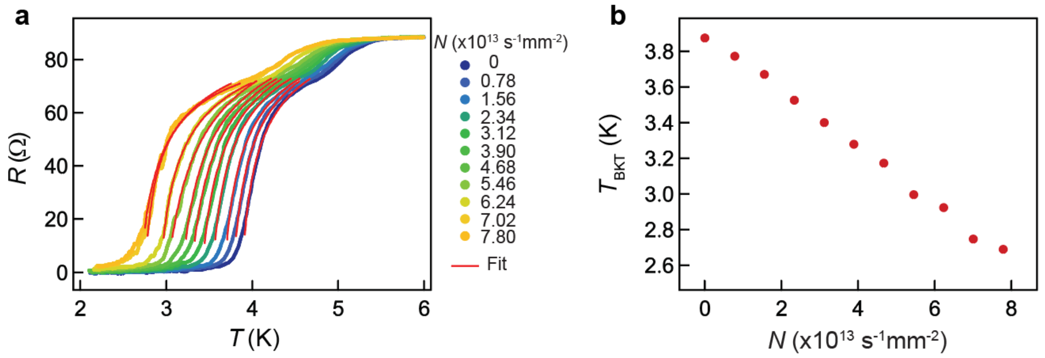

Supplementary Figure 11. Temperature dependent resistance related to BKT physics. (**a**) Sample resistance R of device A1 (AuNPs/hBN/$NbSe_2$) as a function of temperature at different photon fluxes of 532-nm light. The transport measurements are performed with AC current 500nA. The red curves correspond to the fittings using the Halperin-Nelson formula[3]: $R(T) = R_0 \exp[-b(\frac{T}{T_{BKT}} - 1)^{-1/2}]$, where $R(T)$ is the temperature dependence of the resistance, T_{BKT} is BKT transition temperature, R_0 and b are material-specific parameters. (**b**) The extracted BKT transition temperature T_{BKT} as a function of photon flux N.

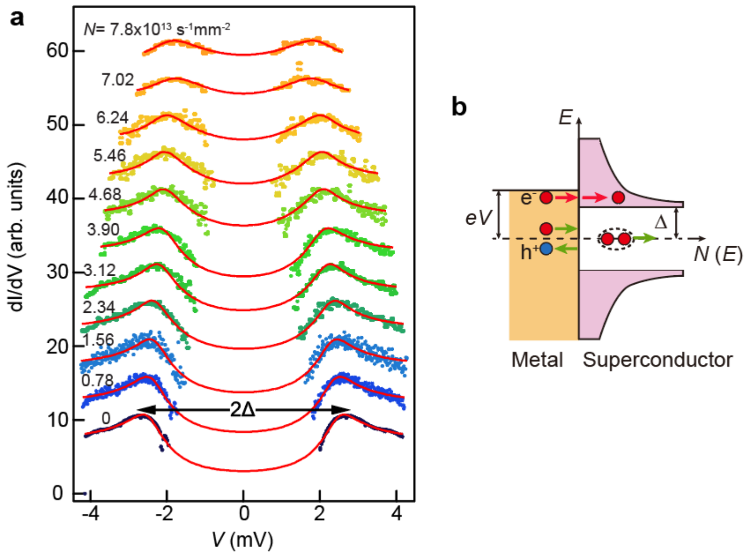

Supplementary Figure 12. Differential conductance spectra of $NbSe_2$ at different photon fluxes and schematic of the electron transport at the normal metal/superconductor interface. (**a**) Differential conductance spectra of $NbSe_2$ at different photon fluxes. The data in **a** are obtained by numerically differentiating the results in Fig. 2a in the main text. (**b**) Schematic of the electron transport at the normal metal/superconductor interface.

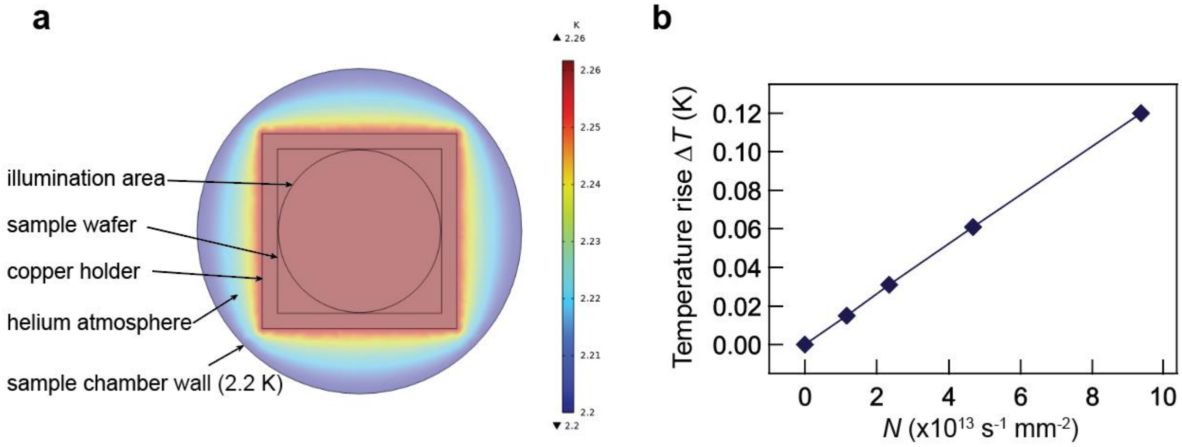

Supplementary Figure 13. Estimation of the temperature rise by COMSOL Multiphysics software. (**a**) Temperature distribution simulation based on the experimental details. Illumination photon flux is 4.68×10^{13} $s^{-1}mm^{-2}$. Assuming all the absorbed photons are converted to heat, a maximum temperature rise of 2.2 K-to-2.26 K is obtained at the sample plane. (**b**) The simulated temperature rise ΔT as a function of photon flux N.

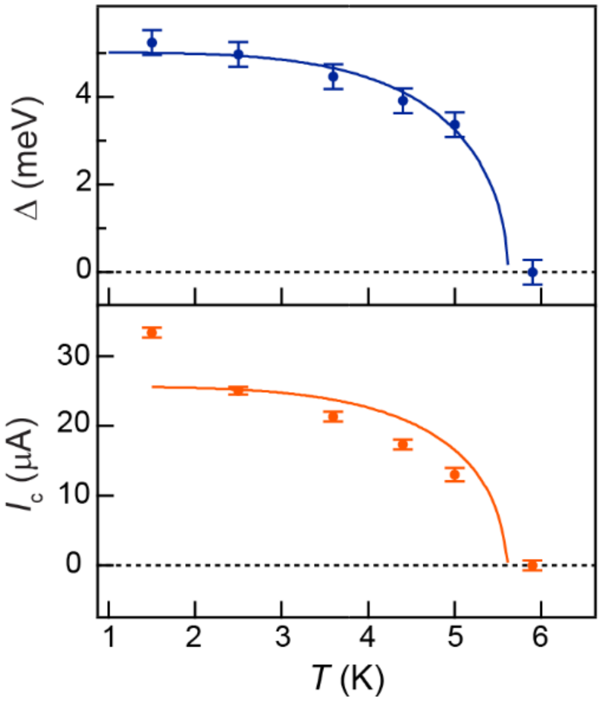

Supplementary Figure 14. Temperature dependences of the superconducting gap Δ and critical current I_c obtained in AuNPs/hBN/NbSe$_2$ device A3. No light illumination is employed. Δ and I_c are obtained by the same Voltage–current (*V*-*I*) characteristics as Fig. 2 in the main text and Supplementary Figure 12. The error bars are obtained by the fitting errors. Temperature dependence of Δ is fitted by the Bardeen-Cooper-Schrieffer (BCS) theory temperature-dependent superconducting energy gap $\Delta(T) = \Delta(0)\tanh 2.2\sqrt{(T_c - T)/T}$, where $\Delta(0)$ and T_c are the zero-temperature superconducting gap and the critical temperature, respectively. The temperature dependence of I_c is fitted by the Ambegaokar-Baratoff (AB) theory[4] $\frac{I_c(T)}{I_c(0)} = \frac{\Delta(T)}{\Delta(0)}\tanh\left[\frac{\Delta(T)}{2k_BT}\right]$, where $I_c(0)$ and k_B are the zero-temperature critical current and Boltzmann constant, respectively.

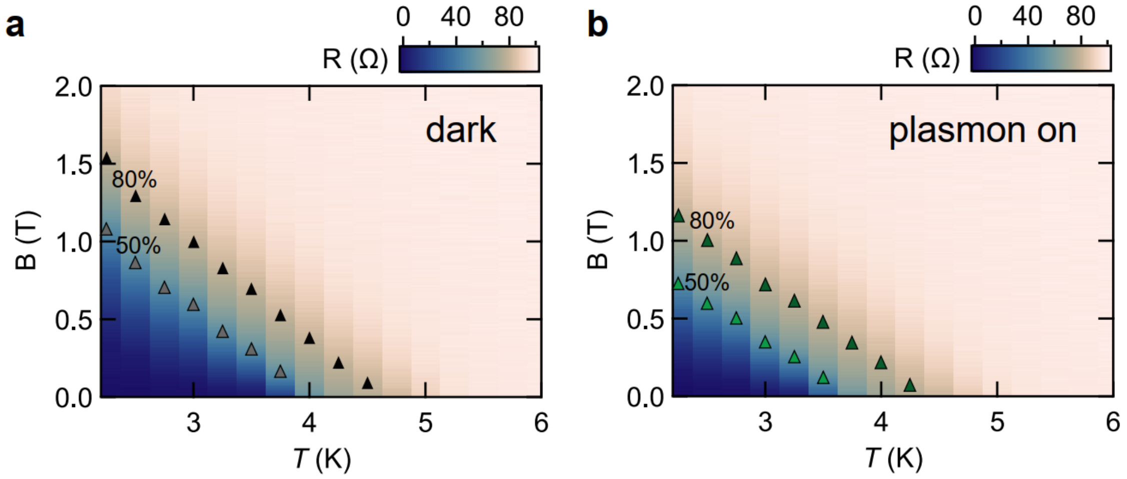

Supplementary Figure 15. Sample resistance of device A1 (AuNPs/hBN/$NbSe_2$) as functions of temperature and perpendicular magnetic field *B* (a) without and (b) with plasmon excitation ($N = 1.56\times10^{13}$ $s^{-1}mm^{-2}$). The grey/light green (black/dark green) triangles are the extracted B_c defined by the field at which *R* reaches 50% (80%) of the normal state resistance. The measurements are taken with two-terminal configuration, same to Fig. 1 in the main text. A contact resistance $R_c = 54.5$ Ω has been deducted.

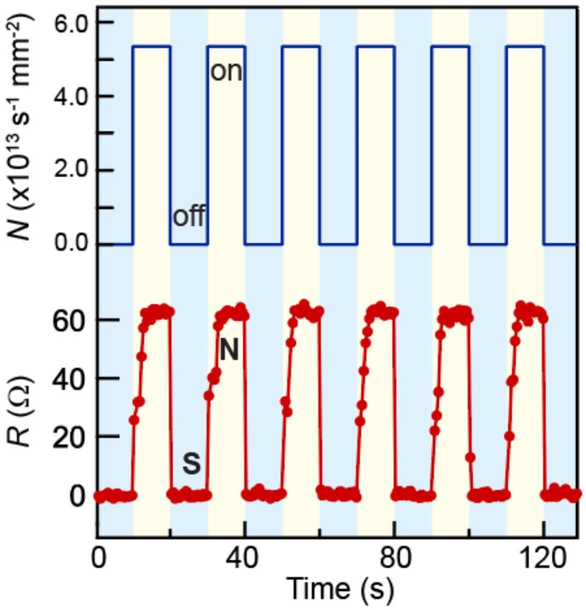

Supplementary Figure 16. Plasmonic superconducting switch operates in low-bias-current mode. Reversible switching between superconducting (S) and normal (N) states of device A1 (AuNPs/hBN/$NbSe_2$) as the plasmon excitation is turned on and off by the 532-nm light. The superconducting switch operates under a low current of 500 nA at a temperature of 3.9 K close to the critical temperature T_c. The measurements are taken with two-terminal configuration, same to Fig. 1 in the main text. A contact resistance R_c = 54.5 Ω has been deducted. Compared to the switching behavior at low temperatures (Fig. 3 in the main text), the switching behavior close to critical temperature T_c shows a slower switching on seconds timescale and asymmetric behavior, i.e., slow OFF-to-ON switching and fast ON-to-OFF switching. At low temperatures (deep inside the superconducting state), the switching behavior depicted in Fig. 3 in the main text is primarily attributed to the coupling between plasmon-induced evanescent field and electrons in $NbSe_2$, which typically occurs in an ultrafast timescale. The small temperature rise induced by the light heating effect (Supplementary Figure 13) does not significantly affect the condensation of Cooper pairs, as the system is deeply inside the superconducting state. However, at temperatures close to the sharp superconducting drop around T_c, the sample resistance is sensitive to the small temperature rise of the light heating effect. In such case, the light heating effect, which commonly involves a gradual heat/energy accumulation process, is likely to be responsible for the slow switching observed at 3.9 K. While the slow OFF-to-ON switching is associated with the gradual heat accumulation process, the ON-to-OFF switching is relatively faster possibly because the accumulated energy can rapidly dissipate into the low-temperature environmental bath. Such asymmetric behavior has also been reported in other superconductor-based devices. For example, the current direction-induced superconducting-normal state transitions in twisted bilayer graphene, as shown in the Supplementary Figure 11 of the Ref. 5.

Supplementary Note 1. Detailed transfer processes of AuNPs

The current study involves the synthesis of octadecanethiolate-passivated gold AuNPs via a two-step, two-phase method[6]. Initially, citrate-stabilized AuNPs with a diameter of approximately 6 nm were created by reducing chloroauric acid in an aqueous solution. Subsequently, these AuNPs were transferred to toluene and coated with octadecanethiol (Sigma-Aldrich) through a modified two-phase method. The octadecane thiolate-passivated AuNPs were then self-assembled into a densely-packed monolayer on a substrate using a dip coating method. During the self-assembly process, the alkanethiol-capped layer's length controlled the interparticle distance, and the ruby-red nanoparticle solution turned purple upon completion due to the AuNPs' hexagonal close-packed array on the substrate's surface. The resultant 2D monolayer of large-area, close-packed AuNPs exhibited uniformity and long-range ordering, allowing the fabrication of compact nanoparticle assemblies over large areas. The diameter of AuNPs is ~ 4 nm, and the thickness of the 1-octadecanethiolate layer is ~1.8 nm (Figure 1).

Supplementary Note 2. Theoretical model of the superconductivity modulation by plasmon

In this section, we develop a phenomenological model to understand the linear suppression of superconducting transition temperature T_c by the resonant excitation of plasmon in gold nanoparticles (AuNPs). The experimental results are summarized in Fig.1 in the main text.

Thermal equilibrium---As demonstrated in earlier studies[7, 8], the thickness dependence of T_c in thin film $NbSe_2$ is well captured by the minimal model that takes into account the intra-layer pairing interaction and inter-layer Josephson scattering. In this model, the interaction Hamiltonian in the pairing channel is given as

$$H_p = -U\sum_{l} c_l^\dagger c_l^\dagger c_l c_l - V\sum_{\langle ll'\rangle} c_l^\dagger c_l^\dagger c_{l'} c_{l'}, \tag{1}$$

where $c_l^\dagger\,(c_l)$ denote electron creation (annihilation) operator in layer l, $\langle ll'\rangle$ denotes that l and l' are nearest neighbor layers, U and V are strengths of intra-layer pairing interaction and inter-layer Josephson scattering, respectively. The linearized gap equation reads

$$\Delta_l = \sum_{l'} \chi_{ll'}\Delta_{l'}, \tag{2}$$

where Δ_l is the order parameter of the l-th layer and χ denotes the pairing susceptibility given as

$$\chi = \begin{pmatrix} U\Pi_0 & V\Pi_0 & 0 & \cdots \\ V\Pi_0 & U\Pi_0 & V\Pi_0 & \cdots \\ 0 & V\Pi_0 & U\Pi_0 & \cdots \\ \vdots & \vdots & \vdots & \ddots \end{pmatrix}. \tag{3}$$

In the above equation, the particle-particle susceptibility Π_0 is defined as

$$\Pi_0 = -\sum_k \frac{f_0(\epsilon_k) - f_0(-\epsilon_k)}{2\epsilon_k} = N_F \int \frac{\tanh\left(\frac{\beta\epsilon}{2}\right)}{2\epsilon} d\epsilon, \tag{4}$$

where f_0 is the Fermi-Dirac distribution function of electrons in the thermal equilibrium state, $\beta = 1/k_B T$, and N_F is the Fermi-level density of states. By equaling the largest eigenvalue of χ to 1, the critical temperature T_c is obtained as[7]

$$T_c = \Theta \exp\left(-\frac{1}{\lambda_{eff}}\right), \tag{5}$$

with

$$\lambda_{eff} = N_F\left[U + 2V\cos\left(\frac{\pi}{L+1}\right)\right]. \tag{6}$$

For the electron-phonon mechanism of superconductivity, Θ is characterized by the Debye frequency of phonon. Based on Eqs. (5) and (6), the effective electron pairing strength λ_{eff} and the resulting T_c depend on the total number of $NbSe_2$ layers L [7, 8].

dynamical equilibrium---Before introducing the model details, we briefly describe the microscopic processes involved in the plasmon-enhanced light-matter coupling. The excitation of plasmon in AuNPs results in a significant evanescent field that decays exponentially away from the AuNPs with a characteristic length of nanometers[9]. This evanescent field can penetrate into the $NbSe_2$ layers placed close to AuNPs, and efficiently excite electron-hole pairs with an energy of ~2.33 eV (532-nm light illumination) in $NbSe_2$. The plasmon-excited hot carriers lose energies through two channels. First, they can rapidly relax to states near the Fermi level via electron-electron scattering within tens to hundreds of picoseconds[10, 11]. Secondly, they can scatter with phonons within hundreds of picoseconds[12], leading to significant nonequilibrium phonon populations. Therefore, upon constant laser illuminating, the system can rapidly relax into a dynamical equilibrium state, ultimately resulting in the redistribution of electrons in $NbSe_2$. The observed plasmon-induced superconductivity modulation in the present system can be interpreted as follows: the plasmon-enhanced electron-hole excitations and the associated phonon repopulations in energies larger than the superconducting gap contribute to the breaking of Cooper pair in the superconducting state, suppressing the superconductivity in $NbSe_2$.

To give a more quantitative analysis, we solve the linearized gap equation by assuming that the normal-state redistribution of electrons is layer-dependent when the system is in a dynamical equilibrium state under constant layer illumination. Specifically, the departure of electron distribution from thermal equilibrium is formulated as

$$\delta f_k = f_k - f_0(\epsilon_k), \tag{7}$$

which can be uniquely decomposed into two sets of $\{\delta f_k\}$ that are, respectively, even and odd with respect to inversion through the local Fermi surface[13]. The even mode also known as energy mode is purely excited from neutral perturbations such as phonons and photons. The odd mode also called charge mode is usually induced by charge perturbations such as tunneling electrons and external currents. For the present system, δf_k is primarily comprised of the energy mode due to the neutral interaction between plasmon-induced evanescent field and $NbSe_2$.

The nonequilibrium distribution function f_k is in general unknown. Nevertheless, it is suggestive to introduce an effective quasiparticle temperature T^* to approximate the redistribution function of the energy mode. Since the plasmon-induced evanescent field decays exponentially into the $NbSe_2$ layers, we may model the parameter T^* as

$$T_l^* = T_1^* \exp[(1-l)\eta], \tag{8}$$

where T_l^* is the effective temperature of the l-th layer, η is a system-specific dimensionless decay coefficient. Based on the above assumptions, the pairing susceptibility for this thermal nonequilibrium multiple-layer system is given as

$$\chi = \begin{pmatrix} U\Pi_1 & V\Pi_2 & 0 & \cdots \\ V\Pi_1 & U\Pi_2 & V\Pi_3 & \cdots \\ 0 & V\Pi_2 & U\Pi_3 & \cdots \\ \vdots & \vdots & \vdots & \ddots \end{pmatrix}. \tag{9}$$

In Eq. (9), the layer-dependent particle-particle susceptibility Π_l is given as

$$\Pi_l = N_F \int \frac{\tanh(\beta_l^* \epsilon/2)}{2\epsilon} d\epsilon, \tag{10}$$

with $\beta_l^* = 1/k_B T_l^*$. Similarly, the superconducting critical temperature is determined by equaling the largest eigenvalue of χ to 1.

Particular case L=2---We first consider a particular system that contains only two layers of $NbSe_2$. By further assuming $U = V$, we have the following linearized gap equation

$$(U + V)\Pi_1 - 1 = V(\Pi_2 - \Pi_1). \tag{11}$$

For the thermal equilibrium state, $\Pi_1 = \Pi_2 = \Pi_0$ and T_{c0} is determined by $(U + V)\Pi_0 = 1$. By inserting this relation into Eq. (11), we have $T_{c0}^2 = T_1^* T_2^* = T_c^2(1 + \delta)(1 + \delta e^{-\eta})$ with $\delta = (T_1^* - T_c)/T_c$. For $\delta \ll 1$, we have

$$T_c \approx T_{c0}\left[1 - \frac{(1 + e^{-\eta})}{2}\delta\right], \tag{12}$$

which is suppressed in a linear behavior as a function of δ. For the present system, it is reasonable to assume the departure from the thermal equilibrium state is proportional to the photon flux number (power of light). Therefore, T_c should be initially suppressed in a linear behavior upon increasing the photon flux number (power of light), consistent with experimental observations in the main text.

Multiple layers of $\boldsymbol{NbSe_2}$---Next, we consider systems comprised of multiple layers of $NbSe_2$, the $T_c's$ of which can be numerically calculated via solving Eqs. (8), (9), and (10). To do so, we adopt the strengths of pairing interactions (U and V) obtained from fitting experimental data[2], namely, $\lambda_0 = N_F U = 1.05$ and $\lambda_1 = N_F V = 0.53$. The decay coefficient η is a prior unknown parameter. In the following calculations, the value of η is chosen to give the best fitting to experimental data.

Supplementary Figure 8a shows the dependence of T_c of three layers of $NbSe_2$ on the nonequilibrium temperature T_1^* by choosing different values of η. The initial stage of T_c suppression exhibits a nearly linear behavior. This linear suppression regime becomes smaller upon increasing η. By further increasing η, the T_c suppression crosses over to another weaker linear suppression regime, as depicted in Supplementary Figure 8a. Supplementary Figure 8b shows the dependence of T_c on T_1^* for different thicknesses (number of layers L) of $NbSe_2$. It is consistent with experimental observation that the suppression of T_c becomes weaker for thicker $NbSe_2$.

To further fit the experimental data, we need to get the knowledge of the relation between the nonequilibrium temperature δT_1^* and the incident photon flux N (power of light). Because the strength of the plasmon-induced evanescent field is proportional to N, a first-order approximation assumes $\delta T_1^* = \lambda N$, where the parameter λ defines the efficiency of driving the top layer of $NbSe_2$ out of thermal equilibrium by the light. Supplementary Figure 9a shows theoretical fitting to the experimental data of T_c as a function of N for $L = 3$.The nearly linear suppression of T_c upon increasing N is well captured, validating the present theory. Moreover, by choosing identical parameters η and λ to those used for $L = 3$ in Supplementary Figure 9a, the layer dependence of the linear suppression coefficient is well predicted by our theory, as depicted in Supplementary Figure 9b, where the coefficient exhibits an exponential decay behavior as a function of L.

Overall, we conclude that the coupling between $NbSe_2$ and the evanescent field induced by the resonant excitation of plasmon drives the electron distribution in $NbSe_2$ out of thermal equilibrium, leading to the suppression of the superconducting critical temperature.

Supplementary Note 3. Differential conductance and BTK model

Here we consider the electron transport in our device as electron transmitting through a normal metal/superconductor interface, as shown in Supplementary Figure 12b. We note that the whole voltage drop V occurs at the interface due to that the total contact resistance is subtracted and eliminated (see Methods). For $|eV| < \Delta$ (2Δ is the superconducting gap), the conductance is dominated by Andreev reflection (green arrows). For $|eV| > \Delta$, the transmission of normal electrons into the electron and hole branches of the quasiparticles in the superconductor (red arrows) contributes to the extra conductance. Therefore, the separation between dI/dV peaks corresponds to the superconducting gap 2Δ. For a more quantitative analysis of the dI/dV spectra, we employ the Blonder-Tinkham-Klapwijk (BTK) model[14], which includes both the single quasiparticle transmission and the Andreev reflection with a finite transparency of the interface. The BTK conductance at zero temperature is given as $\sigma(E) = \tau_N \frac{1+\tau_N|\gamma(E)|^2+(\tau_N-1)\left|\gamma(E)^2\right|^2}{|1+(\tau_N-1)\gamma(E)^2|^2}$, where τ_N is the transparency of the barrier, $\gamma(E) = \frac{E-\sqrt{E^2-\Delta^2}}{\Delta}$ and E = eV+iΓ with Γ denoting the broadening parameter. The fittings are shown as red curves in Supplementary Figure 12a. The extracted superconducting gap Δ as a function of photon flux N is shown in Fig. 2b in the main text.

Supplementary References

1. Xi, X. & Carr, G. L. A THz time-domain susceptibility for superconductors including strong-current effects. *Supercond. Sci. Technol.* **26**, 114001 (2013).
2. *Near-field optics and surface plasmon polaritons*. Springer: Berlin ; New York, 2001.
3. Halperin, B. I. & Nelson, D. R. Resistive transition in superconducting films. *J. Low Temp. Phys.* **36**, 599-616 (1979).
4. Barone, A. P., G. *Physics and applications of the Josephson effect*. Wiley, 1982.
5. Díez-Mérida, J. et al. Symmetry-broken Josephson junctions and superconducting diodes in magic-angle twisted bilayer graphene. *Nat. Commun.* **14**, 2396 (2023).
6. Lin, M.-H. Chen, H.-Y. & Gwo, S. Layer-by-layer assembly of three-dimensional colloidal supercrystals with tunable plasmonic properties. *J. Am. Chem. Soc.* **132**, 11259-11263 (2010).
7. Schneider, T. Gedik, Z. & Ciraci, S. Transition temperature of superconductor-insulator superlattices. *Europhysics Letters* **14**, 261 (1991).
8. Xi, X. et al. Ising pairing in superconducting $NbSe_2$ atomic layers. *Nat. Phys.* **12**, 139-143 (2016).
9. Montaño-Priede, J. L. & Pal, U. Estimating near electric field of polyhedral gold nanoparticles for plasmon-enhanced spectroscopies. *J. Phys. Chem. C* **123**, 11833-11839 (2019).
10. Parham, S. et al. Ultrafast gap dynamics and electronic interactions in a photoexcited cuprate superconductor. *Phys. Rev. X* **7**, 041013 (2017).
11. Yang, X. et al. Terahertz-light quantum tuning of a metastable emergent phase hidden by superconductivity. *Nat. Mater.* **17**, 586-591 (2018).
12. Yang, X. et al. Nonequilibrium pair breaking in $Ba(Fe_{1-x}Co_x)_2As_2$ superconductors: evidence for formation of a photoinduced excitonic state. *Phys. Rev. Lett.* **121**, 267001 (2018).
13. Tinkham, M. *Introduction to superconductivity*, 2nd edn. McGraw Hill: New York, 1996.
14. Daghero, D. & Gonnelli, R. S. Probing multiband superconductivity by point-contact spectroscopy. *Supercond. Sci. Technol.* **23**, 043001 (2010).
